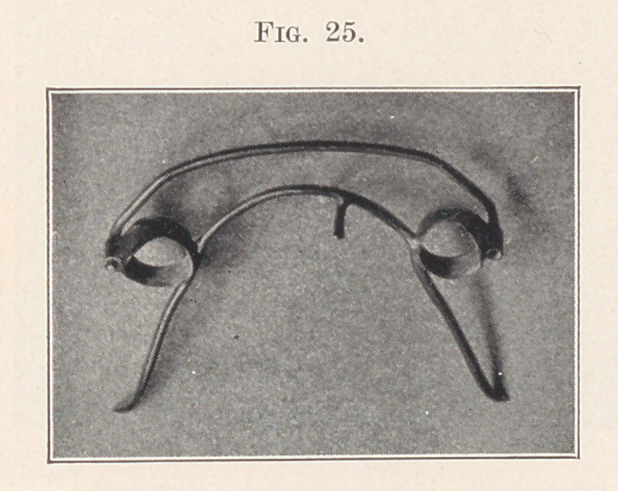# Some Thoughts Regarding Methods, and a New Appliance for Moving Dislocated Teeth into Position

**Published:** 1904-07

**Authors:** George C. Ainsworth

**Affiliations:** Boston, Mass.


### International Dental Journal.

Vol. XXV. July, 1904. No. 7.

### Original Communications.[1]

### SOME THOUGHTS REGARDING METHODS, AND A NEW APPLIANCE FOR MOVING DISLOCATED TEETH INTO POSITION.[2]

BY GEORGE C. AINSWORTH, D.D.S., BOSTON, MASS.

It has been suggested to assume that my audience is totally unfamiliar with everything connected with this subject except that they are dentists.

I wish to say that the time at my command since accepting the invitation to appear before you this evening has not been sufficient to accomplish all that I desired, more especially since some of the pictures which will be shown have necessarily been made from old models that were made with no thought of the importance of perfection, and simply for study models. Some of the appliances, too, in their application to the models are not perfect in their adaptation, but simply gotten up to demonstrate the theory of their application.

It is my purpose, this evening, to place before you *three* appliances, all of which I have been using for some time with much satisfaction, and while the principles involved are not new, the

[1] The editor and publishers are not responsible for the views of authors of papers published in this department, nor for any claim to novelty, or otherwise, that may be made by them. No papers will be received for this department that have appeared in any other journal published in the country.

[2] Read before The New York Institute of Stomatology, March 1, 1904.

manner of applying them *is*, so far as I have been able to learn. The first is a self-acting spreading appliance, the second is an inclined plane for jumping the bite and adjusting the occlusion, while the third is a simple retaining appliance.

To appreciate the increasing need of orthodontic services, one has but to observe the faces of the rising generation we meet every day. The causes are many and the conditions alarming. Indeed, the patient whose teeth cannot be improved by the orthodontist seems the exception rather than the rule.

It also seems to be the opinion of those best able to judge, that this work should be largely done by the specialist. Certain it is that the successful orthodontist must be a man of large resource, —in art, in mechanics, and in patience,—for there is no hard and fast rule for the solution of all cases, and while we may classify them in general terms, there still remains an individuality which necessitates an independent solution of the case in hand according to its merits.

Nevertheless, while conditions remain as they are the average dental practitioner, especially in the smaller districts, will be called upon to do more or less of this work. It then becomes a duty for us to be keenly alive to any methods which may promise the greatest good with the least amount of suffering to the patient, as well as the least draft upon the time of the operator.

We should study to simplify methods and appliances, to minimize pain, and to economize time. Along these lines I have expended much time and thought to produce something that should be self-acting, requiring little attention, and which should interfere as little as possible with the ordinary functions of the teeth and mouth, not forgetting cleanliness, which presupposes simplicity and a due regard for one's appearance.

Teeth are made irregular by pressure, of one sort or another, and without pain. Why, then, should we not expect to make them regular by pressure without pain? In the first instance, the pressure is against nature, while in the second it is in accordance with her design. It seems, then, to be a question of applying the necessary force to move them in a proper way.

We have two kinds of force at our command,—the intermittent, as applied by the screw, and the constant, as applied by the spring, the lever, the ligature, and the elastic.

The claim has been made in favor of the screw pressure, that force thus applied was attended with less soreness and consequent discomfort; that there should be periods of rest between the periods of movement such as might be obtained by turning the screw each day; that the force having spent itself, the tooth rested until the next turn of the screw. That claim, it seems to me, is not borne out by facts; and while it undoubtedly is true that force applied by an elastic band or rubber wedge is attended with more soreness and consequent pain, it seems to be owing to that peculiar quality of elastic force as applied by rubber, and which differs from force as applied by a spring. What seems to be tolerated most kindly is a firm, gentle pressure, be it intermittent or otherwise. When a tooth first starts it is usually attended with slight tenderness, which, as a rule, quickly subsides without a letting up of that force; then, if the force is continued in a reasonable way, all goes well.

It is idle, perhaps, to talk about pleasure to the patient in having teeth regulated. Nevertheless, there are different degrees of comfort, and it is to obtain the maximum that we are striving. To do this the work had best go on slowly. The effort to accomplish an extensive piece of work in orthodontia in a remarkably short time seems to me to be ill advised, and not likely to be attended with as satisfactory results as it would have been had the change been conducted more gradually. All these changes are accomplished by force, which, if applied too suddenly, or too severely, renders nature antagonistic and rebellious, as evidenced by pain, heat, redness, and swelling,—she recognizes an enemy; whereas, if the force is applied gradually and gently, she looks upon you as a friend trying to help her, and takes kindly to your efforts, falling into line with all the assistance of which she is capable.

Again, where teeth are moved slowly they are less liable to be drawn out of, or away from, their tissue environment,—a matter of much importance, particularly when elongating teeth.

We come now to a consideration of the self-acting spreading appliance, which may have a double action,—*i.e.*, it can be so adjusted as to spread the arch and, when desirable, retract the incisors at the same time, or, by the addition of ligatures, the front teeth may be moved forward or elongated. In accomplishing the first two, it is entirely self-acting and requires very little attention, allowing the absence of the patient two, four, or even eight weeks at a time, yet its action is under the control of the patient at all times.

In the second instance, where ligatures are employed it requires closer attention, but where wire ligatures are used, even those allow the absence of the patient for some days. Yet I find many occasions to use silk ligatures, even though they require more constant attention, for the sake of cleanliness.

This appliance, in its simplest form, is composed of three members (Fig. 1, A),—two anchors and a wire spring, while the compound form has two springs. Each anchor is made up of three pieces,—a piece of seamless tubing, with 30-gauge walls, of suitable size and length to be fitted to the tooth chosen for anchorage, after the manner of forming a band for a gold crown. To this is soldered, on the palatal side and at right angles with the band, a piece of 16-gauge wire running along the border of the arch, with a bearing on and of sufficient length to engage all of the teeth to be moved on that side; while on the buccal side of the anchor band is soldered a short piece of 16-gauge seamless tubing running parallel with the band, to receive the end of the spring-wire,—the active principle of the appliance. These anchors, when completed, are adjusted to the teeth selected, and cemented firmly into place (Fig. 1, B)—one on either side of the arch, after which the two ends of the spring wire, bent at right angles to itself, are sprung into the tubes provided for them (Fig. 1, C). The inside bar is designed to move the bicuspids and molars as a unit without the aid of ligatures.

This case seems to be an unusually favorable one for this appliance, which in its operation has a double action. The sides of the arch are to be equally expanded and the incisors moved in (Fig. 1, D). The teeth to be moved out include the cuspids, the bicuspids, and the first molars. The spring, being adjusted to bear firmly on the labial surface of the incisors, presses those teeth in as the side teeth move out. The teeth chosen for attaching the anchor bands should be midway between the two points of resistance, and this perhaps more often fails on the first bicuspid than otherwise, though sometimes it may be the second bicuspid, or even the first molar.

My estimate of the central point of resistance in this case is the first bicuspid, the cuspid and first molar being the extreme points. You will readily see that if the pressure be applied in the molar region it would not expand the arch at the cuspid, which tooth offers marked resistance. If, however, during the operation you

Fig. 1.

A B

C D

Fig. 2.

Fig. 3.

FIG. 4.

FIG. 5.

Fig. 6.

find your judgment has been in error for any reason, it is not a difficult matter to change the attachment to a more favorable location.

The time required to accomplish the desired change depends upon the age of the patient and the adjustment of the spring. I should not hesitate to dismiss this patient for two months after adjusting the appliance, but where convenient I would look at it about once in two weeks, always directing that if anything seems wrong, or soreness develops, to report at once. The spring, as you see, can be readily removed and readjusted by the patient.

Fig. 2 shows another case for the simple appliance. All the teeth seem in satisfactory relation excepting the second upper bicuspid on the right side, which occludes inside the arch.

The appliance was adjusted here to pit the resistance of the five teeth on the left—all the teeth from the cuspid to the second molar—against the resistance of the one displaced tooth on the right.

In eight weeks the tooth had moved to the position as you now see it (Fig. 3), with practically no attention or change, and, if I may believe the patient, without any pain or inconvenience whatever. Undoubtedly, had the tooth not been locked in by the approximating teeth, it would have moved to position in half the time. The small wire on the inside was put on as a precaution, lest the tooth should start suddenly, when it had progressed far enough to throw off the lateral resistance, and come out too far. On its completion the appliance was removed and a retainer, consisting of a simple band, cemented to the tooth, to the buccal side of which was soldered a piece of gold wire, similar to the one shown on the palatal side, the ends of which rested against the buccal surfaces of the approximating teeth.

Fig. 4 shows a case where the simple appliance would be appropriate, but where I should choose the second bicuspid for anchorage of the bands and place the spring-wire tubes at the antero-buccal surface.

Fig. 5 shows a case now under treatment where the slight contraction of the arch has forced the centrals and cuspids to assume a position outside of the arch, or perhaps, more correctly speaking, the bicuspids are inside of the arch. The simple appliance has been adjusted, anchored to the first bicuspids, and the arch is gradually spreading. I see the patient once a week, just to keep an eye on it, but as yet have made no change. In eight weeks, I should judge, the expansion will be sufficient to allow the turning of the cuspid teeth into position.

I have been asked to explain why I commenced work on the upper teeth instead of the lower. It is not always easy to give a lucid explanation of the reason for doing a certain thing, although one follows the dictates of his judgment—*instinct,* perhaps. It is true, nature begins her work in the adjustment of her masticatory apparatus by the development of the lower teeth first, but the conditions under which she does it are different from those presented to us for correction. She moves the teeth upward and downward on a line with the axes of the tooth, while we are called upon to move them sidewise. Again, the lower teeth are inferior in size and stability to the upper, which, when fully developed, have a larger controlling influence, and it seems to me as a rule easier to expand the larger arch first after which the smaller is more easily adjusted to it, especially as the upper jaw is stationary, while the lower is not. In other words, there are limitations to the possibilities of movement or adjustment in the upper jaw that do not obtain in the lower.

It is, however, perfectly practicable with this appliance to carry both upper and lower along simultaneously, sometimes a matter of much importance, since it lessens the number of visits and expedites the work. Indeed, there are many cases where inconvenience and discomfort are lessened by having the upper and lower carried on together.

Fig. 6 shows another case,—that of a nervous, excitable, young lady thirteen years of age. The parents dreaded the commencement of the operation, lest it upset her entirely. The simple appliance has been on now four weeks, with perfect comfort and happiness; in fact, I think she rather enjoys it. Her visits are made once a week in company with a young lady friend undergoing the same sort of an operation, and there is invariably a controversy between them as to which shall occupy the chair first. My attendant usually settles it by having them draw lots. I speak of this merely to show that the work is not always dreaded.

You will notice (Fig. 7) that the molars on one side occlude inside the arch, while the bicuspids are not yet far enough advanced to determine exactly where they would come. The appliance is anchored to the first molars, while the inside bar is so arranged

Fig. 7.

Fig. 8.

Fig. 9.

A B

as to guide the bicuspids out into a proper position. During the four weeks the arch has expanded three-sixteenths of an inch.

Of course, there is nothing remarkable in what is being accomplished in most of these cases, excepting the satisfaction of seeing them progress so favorably without laborious and painful attention. The gums are clean, healthy, and comparatively free from irritation.

Fig. 8 represents the teeth of a lady from forty-five to fifty years of age, and introduces the addition of another spring-wire and its attachment to the simple appliance, making a compound, self-acting appliance which is applicable to those cases where *much* pressure is required at both the cuspid and molar region. The longer spring, acting on the molars, may be adjusted high up under the lip entirely out of sight, while the shorter one, acting on the cuspids, may be adjusted lower down at the pleasure of the operator.

All the teeth are present excepting the lower second molar on the right and the four wisdom-teeth (Fig. 9, A). The upper bicuspids and molars occlude inside the arch on both sides, the front teeth badly bunched, giving to the mouth a decidedly unpleasant expression, the lips closing over them with difficulty. The lower arch is of normal width, but the teeth are somewhat out of position in the left cuspid region, that tooth being, perhaps, one-sixteenth of an inch short.

The simple, one-spring appliance was put on this case in June, with the longer wire, as here shown, adjusted to the molar bands, while the appliance here shown represents what I would use were I to conduct the case again. If I remember correctly, I saw the patient three times before I went on my usual August vacation, but made no change in the appliance. On my return, early in September, I found the arch widened at the molar region fully one-half inch (Fig. 9, B), with the assurance from the patient that she had suffered no inconvenience whatever; in fact, she said she could not believe for a long time that it was accomplishing anything, it was so comfortable. The arch was spread at the cuspid region by a jack-screw running from cuspid to cuspid on the palatal side, after which the alignment was accomplished by the well-known arch band anchored to the molars. Fig. 10 shows the case completed.

You will observe that the tubes on the molars (Fig. 8) run high up opposite the ends of the roots. This was for the purpose of applying the expanding pressure in such a way as to move the roots and process at that point, the long ends of the spring-wire being bent out at such an angle as to apply force *there* when inserted in the tubes. It is also perfectly practicable to insert the wire the other way,—*i.e.*, at the top of the tubes,—and thus apply the force high up, overcoming the tipping tendency sometimes met with in other appliances.

Thus you will see that this appliance offers the possibility of many variations through additions and modifications, which unfold every day in our experience, to solve the problems as they are presented.

Figs. 11 and 12 show a case where the inside bar is used in connection with the ordinary arch band. The cuspids and bicuspids are to be moved out a little, while the front teeth are to be elongated and brought in.

The objection to the self-acting appliance here is the limited downward pressure obtainable with the spring-wire attached to the first bicuspids, besides the possibility of moving those teeth forward while in the act of pressing the front teeth in. If we anchor to the molars we shall not spread the arch at the cuspids and bicuspids without the addition of several ligatures, around those teeth. The same objection applies to the ordinary arch band, anchored to the molars with nuts at the distal ends of the tubes, but by combining the inside bar with this appliance we do away with all such ligatures, substituting one between the cuspid and first bicuspid on either side connecting the inside bar with the spring of the outside band. This will move those teeth out and not materially interfere with the downward spring of the arch band.

The inside bar soldered to the anchor band around the molar counteracts the tendency to the tipping of that tooth, which might be experienced by raising the arch band up and ligating to the front teeth.

The following case is shown, not because of its connection with any appliance presented, but for the reason that it is in some respects quite remarkable.

The gentleman presented at the age of forty-nine. The only contact between the upper and lower teeth was on the right side, between the second molars, which, instead of meeting each other squarely, passed by each other, wearing an inclined plane on either

Fig. 10.

Fig. 11.

Fig. 12.

Fig. 13.

Fig. 14.

A B

Fig. 15.

Fig. 16.

tooth (Fig. 13). The jaws closed much beyond the normal point, giving a most unpleasant expression to the face.

One molar and lateral are missing on the upper left side, and on the right side two molars, one bicuspid, the cuspid, and the lateral, seven teeth in all (Fig. 14, A). In consequence, the contraction of the jaw has been such that all the upper teeth strike well inside the lower arch, the incisors fully one-half inch.

On the lower jaw every tooth back of the first bicuspid on the left side had been extracted, and the right first and second molars were also missing. The question asked by the patient was, "Can you do anything for me, or must I be turned out to pasture to die like an old horse? I cannot chew as I am."

Three months' study of the case and models was necessary before a possible solution of an apparently hopeless case was determined upon. Artificial plates alone were out of the question, because of the great discrepancy in size of the two arches.

It was decided to attempt to widen the upper arch in order to bring the molars and bicuspids as nearly as possible over the lower, since every particle of movement in that direction would be a distinct gain; and to move the incisors as far forward as might be to improve the appearance, though it seemed absolutely out of the question to obtain an occlusion of the incisors.

In four months the result was beyond the most sanguine expectations entertained at the start, and a temporary retainer was inserted for the summer (Fig. 14, B).

In the fall a combined bridge and retainer in one continuous piece was made for the upper, using as abutments the molar and first bicuspid on the left side and the molar and second bicuspid on the right, swinging the missing intervening teeth on as dummies. The missing teeth on the lower jaw were supplied on a partial gold clasp-plate.

The result is a good masticating contact on either side back of the cuspid teeth and a reduction of the antero-posterior distance between the incisors to one-eighth of an inch (Fig. 15).

### MAKING THESE APPLIANCES.

In making these appliances ordinary German silver is used for all except the spring-wire, which is eighteen per cent. nickel especially drawn for the purpose, with the maximum amount of spring obtainable consistent with toughness. If too hard, it is apt to break and cause annoyance. This I have obtained of the Holmes, Booth & Haydens Company, of Waterbury, Conn. The tubing, of various sizes, is seamless drawn, and especially made for me by J. Briggs & Sons' Company, 65 Clifford Street, Providence, R. I.

Ordinary plate, of German silver or gold, may be used, with platinized gold wire for the spring, if one prefers, but I have found the German silver to answer every purpose, and in some respects it is superior. Seamless drawn tubing possesses a marked advantage, particularly when soldering the small tubes to the anchor bands. The appliance when completed is gold-plated.

In making the appliance the method of procedure is as follows: After cutting off a piece of seamless tubing of the proper size, anneal it, and with a pair of contouring pliers form it into a sort of barrel-shaped cylinder; next, with a small pair of scissors cut away a part of one end to approximately correspond to the gum line around the tooth; then gradually work it up into position, perhaps a little under the gum, take an instrument and mark around at the gum line; also at the top, so that there shall be a small projection left after trimming to turn over the edge of the tooth into the sulci, giving the band a firm seat when finally cemented to place. After trimming, the two bands are placed in position and a *plaster* impression taken; *this,* in my judgment, is important, as accuracy counts for much when one comes to set the appliance.

As a rule, it is reasonably possible to force a band of 30-gauge walls between the teeth, but sometimes, as an aid, I draw in some sort of a wedge for fifteen minutes or an hour, as the case may be. A rubber wedge works well for that length of time. Then, again, the edges of the band may be thinned a little and smeared with vaseline; one band may be crowded in a trifle and left while proceeding with the other. In short, a variety of expedients may be resorted to, that will be suggested to the mind of the resourceful man.

After the impression has been removed from the mouth the bands are carefully taken off and replaced in the impression, and the usual detail of making the model gone through with, the bands appearing on it exactly as they will stand in the mouth. Proceed then to adjust the palatal wires to fit the model as desired, being *particularly* careful, if intending to move the cuspids out, to turn that end of the wire well up under the gum in such a way as to engage that tooth above the bulge of enamel; otherwise, when the spring pressure is applied that end of the wire will slide down the incline plane surface of the cuspid instead of moving the tooth, resulting in a troublesome elongation of the tooth banded.

When these wires have been properly fitted, hold them in position by pieces of binding wire passed around them and through the model, twisting the ends till taut.

Next, to adjust the small tubes for receiving the ends of the spring-wire, pass an ordinary pin through them into the model in such a way as to hold them in position while being soldered.

Next, bur out a little of the plaster from within the anchor band opposite the points where the solder is to flow, and proceed to the soldering, which is done on the model.

The next step is to remove, finish, polish, and gold plate.

The labial spring-wire is usually fitted into position after the anchors are placed in the mouth, sometimes before cementing, sometimes after. If the front teeth are to be moved in and spaces closed up, the wire is adjusted to bear as firmly as possible on those teeth; if the front teeth are to be moved out, the wire is adjusted to stand out a bit to admit of the ligatures doing their work. Under favorable conditions, the fitting of the anchor bands and taking the impression can be accomplished in an hour.

### PLATING PLANT.

Some sort of a gold-plating plant seems an important auxiliary to the dental equipment of to-day. A simple one is made up of a glass jar of convenient size with a cover to prevent evaporation and exclude dust, containing a fluid composed of thirty grains of chloride of gold, sixty grains of cyanide of potassium, and one-half pint of distilled water, operated by a single-cell Sampson battery. A piece of pure gold is attached to the carbon wire and suspended in the fluid, while the article to be plated is attached to the zinc wire and likewise suspended, care being taken that the gold and the article to be plated do not come in contact while in the solution.

In place of the Sampson cell, the wires may be connected with the ordinary electric lighting supply, in which case the current is run through a series of lamps to reduce it.

In the latter case the piece to be plated is left in the solution perhaps three minutes, when it is taken out and polished; this process is repeated several times according to the amount of plate desired.

ADVANTAGES.

In summing up the advantages of this appliance, its *simplicity* seems to stand out as a key-note far and above everything heretofore in use. Being simple, it is *cleanly,* doing away largely with ligatures, *always painful to apply and uncomfortable to wear.*

It is *effective,* because it is worn twenty-four hours every day, and the power is applied directly to the teeth to be moved. It interferes as little as possible with the ordinary functions of the teeth and mouth, besides minimizing the deleterious effect sometimes noticed from wearing a more complicated appliance.

It is automatic in its work, and may be adjusted so as to have a double or, by the addition of ligatures, a triple action,—*i.e.,* it will spread the arch, move the front teeth in, and elongate at the same time.

As it is automatic in its action, it requires less attention, and consequently produces less inconvenience and pain, which should lend value to our services.

It is equally applicable to the upper or lower teeth and may be used on both simultaneously.

It is conveniently adjusted to bring pressure to bear on the roots and alveolus, and thus has a tendency to overcome the outward tipping of the anchor teeth sometimes encountered in spreading the arch.

And lastly, it is not unsightly, since it admits of greater cleanliness.

INCLINED PLANE.

The next appliance shown is an inclined plane fixed to the teeth, for "jumping the bite" and adjusting the occlusion.

It sometimes happens in regulating teeth that the lower jaw is all at sea as regards an occlusion of the teeth. It is equally bad in two or three different positions. It becomes then a question of compelling one, and only one, closure in order to give the teeth a chance to adjust themselves to each other after nature's design.

It is particularly applicable where there is excessive overbite of the front teeth, such as usually accompanies a case of thumb-sucking.

Fig. 16 shows such a case, after the upper teeth had been brought into an approximately correct arch relation. An attempt was made to establish a correct occlusion by constructing gold

Fig. 17.

Fig. 18.

Fig. 19.

Fig. 20.

crowns for the upper sixth-year molars with grinding surfaces corresponding to those on the lower teeth when in correct relation, thus allowing the twelfth-year molars, which were just beginning to show through the gum, to erupt into their proper relation. Thus far everything seemed satisfactory, but the gold crowns being removed, observe what happened. The patient instead of adopting the correct occlusion, just shown in the last picture, found the one now on the screen more agreeable and effective, thus defeating the attempt to correct the overbite (Fig. 17).

The obvious objections to an inclined plane in connection with a plate led to the application of the same principle involved in a plane soldered to bands encircling the upper centrals and to lugs engaging the cutting edges of the laterals (Fig. 18), to prevent undue depression of the centrals. This resulted in establishing the correct occlusion shown in Fig. 19.

This appliance may seem at first an uncomfortable one to wear, but this young man affirms that after the first day or two he paid no attention to it, and like reports come from similar cases.

This was the first appliance of the kind I made. I now have them made with a round wire lug to rest over the end of the lateral, as more cleanly and less likely to injure the tooth, and for the same reason have the gold dressed away from the palatal side of the laterals as much as possible to avoid contact. The bands on the centrals cover nearly the whole palatal surface and are firmly cemented on. The inclined plane surface is made from a fairly heavy piece of platinized gold plate.

Fig. 20 shows a case of a young man, twenty-one years of age, where the overbite completely covers the lower front teeth; the lower incisors badly bunched; one central having been extracted. Notice the discrepancy in the length between the front and back teeth on the lower jaw (Fig. 21, A). Both arches were spread, the lower front teeth were somewhat improved and an inclined plane adjusted, as in the previous case. This not only held the teeth apart and facilitated the regulation of the lower front teeth, but it depressed the upper and lower front teeth in their sockets, while the back teeth were free to elongate and meet each other under natural conditions. The final result is seen in Fig. 22.

The time required to produce the desired change varies from perhaps four to twelve months, according to the age of the patient and the amount of elongation to be accomplished.

Some of the teeth to be lengthened in the first case were banded with thin gold, pin-heads being soldered to the bands in such a way that an intermaxillary elastic band could be attached at night and produce an elongating pull, but this was soon abandoned as unnecessary.

The inclined plane need not, as a rule, be as wide as the one shown, and may be reduced as the work progresses. This appliance sometimes facilitates the work of the automatic spreading appliance, and may be worn in conjunction with one of those appliances on the lower teeth. It may also be incorporated with a retainer for the upper teeth such as shown in Fig. 23.

The advantages of this appliance are, first, its simplicity—no ligatures or rubbers required. It is cleanly and effective, automatic in its action, and requires no attention from either patient or operator after it is adjusted. It is not painful, and the patient makes less complaint than the parent.

### RETAINING APPLIANCE.

The next, and last, appliance to be shown is a simple retaining appliance, designed to securely hold the teeth after regulating until they have become fixed in their new position.

The requirements in such an appliance are *security, cleanliness, comfort, appearance.*

The appliance here shown (Fig. 24) combines all these to a marked degree. Like the self-acting expanding appliance (which is an outgrowth from the retaining appliance), it is composed of anchor bands with small tubes attached to the buccal sides, into which are inserted the ends of a labial wire, bent at right angles to itself. The inside wire differs from that of the self-acting appliance in that it is continuous around the arch, thus holding all the teeth that may have been moved out, while the labial wire holds the front teeth in.

This appliance is securely cemented into place and worn without discomfort.

An important and pleasing feature is the removability of the labial wire, as it sometimes happens that a young lady wishes to avoid its appearance for an evening. The wire is readily taken out and readjusted by the patient. In cases where the front teeth have not been moved in, it can be dispensed with altogether, otherwise the appliance is not particularly conspicuous.

Fig. 21.

Fig. 22.

Fig. 23.

Fig. 24.

Fig. 25.

The labial wire is usually smaller than in the self-acting appliance.

Additions may be made to this appliance, as, for instance, when a tooth has been rotated, it may be banded and the band soldered to the inside wire, thus becoming a part of the retaining appliance.

And again, when it is desired to tip a front tooth or teeth inward, moving the apex of the root outward, spurs may be soldered to the inside wire at right angles to it in such a way as to rest on the palatal or lingual surface of the root high up under the gum, exerting, when adjusted, an outward pressure, while the labial spring-wire is adjusted to bear hard on those teeth well down towards the cutting edge (Fig. 25). The result is obvious.

This completes what I have to show this evening, and while it may not all be new to you, I hope some of it is. It has been my desire to make the subject clear. If I have not, I shall be glad to do so later. And if any of you can derive the satisfaction from the use of these appliances that I have, it will be an added source of pleasure to me.

## Figures and Tables

**Fig. 1. f1:**
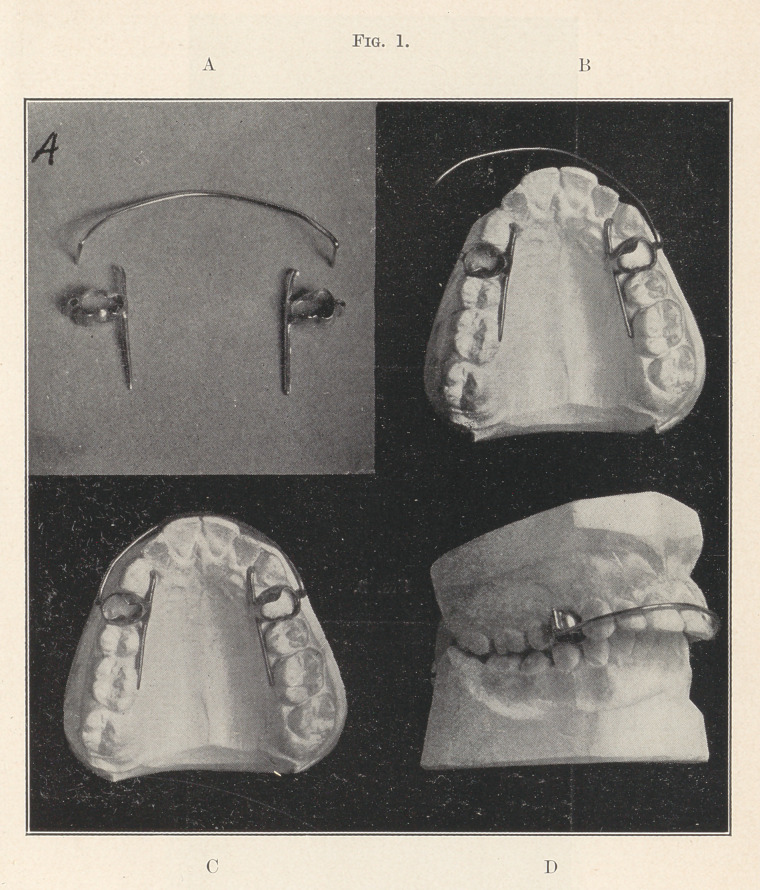


**Fig. 2. f2:**
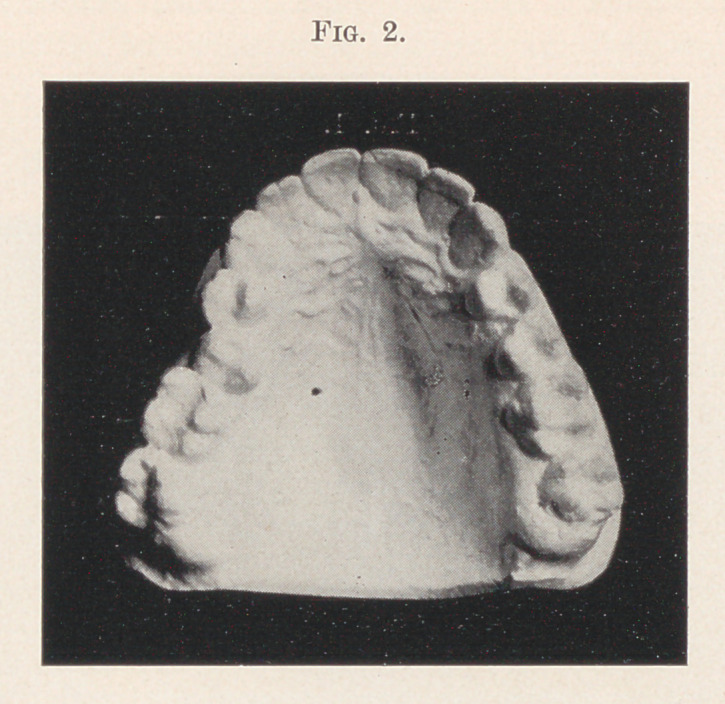


**Fig. 3. f3:**
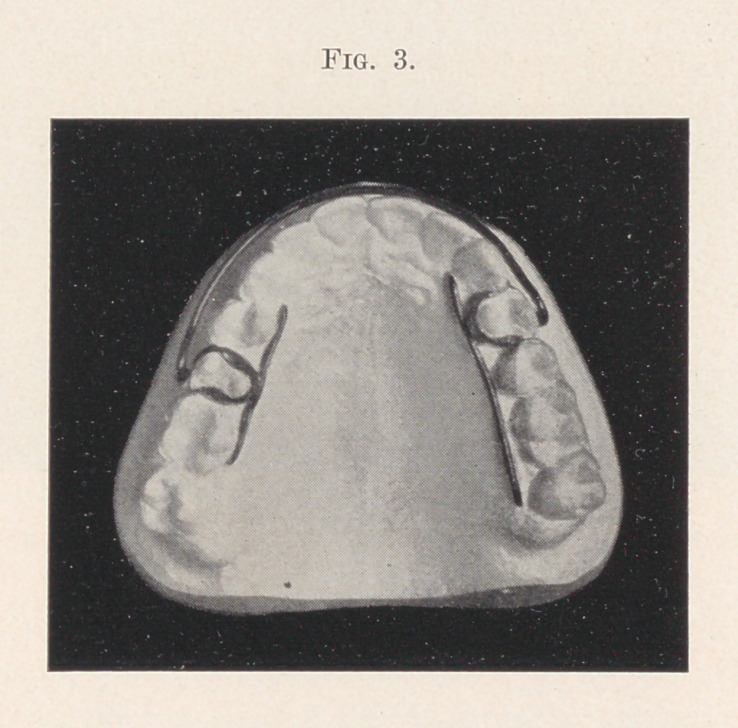


**Fig. 4. f4:**
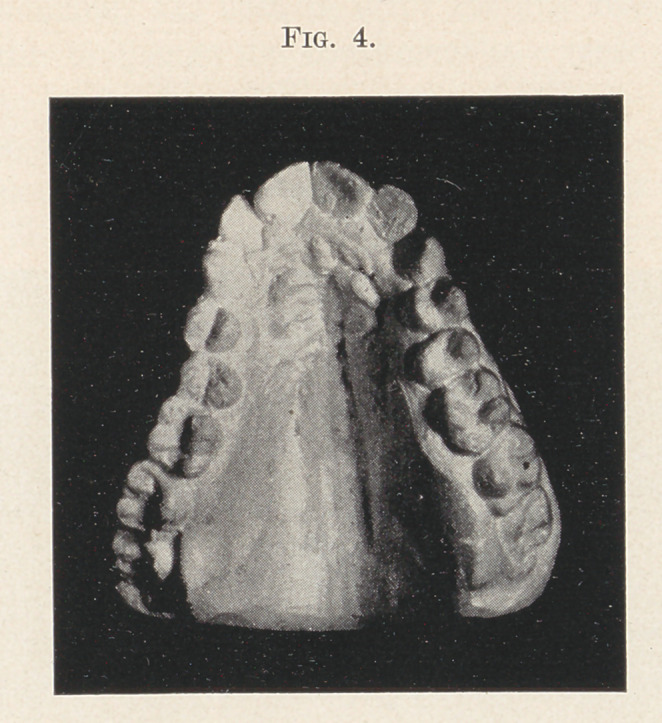


**Fig. 5. f5:**
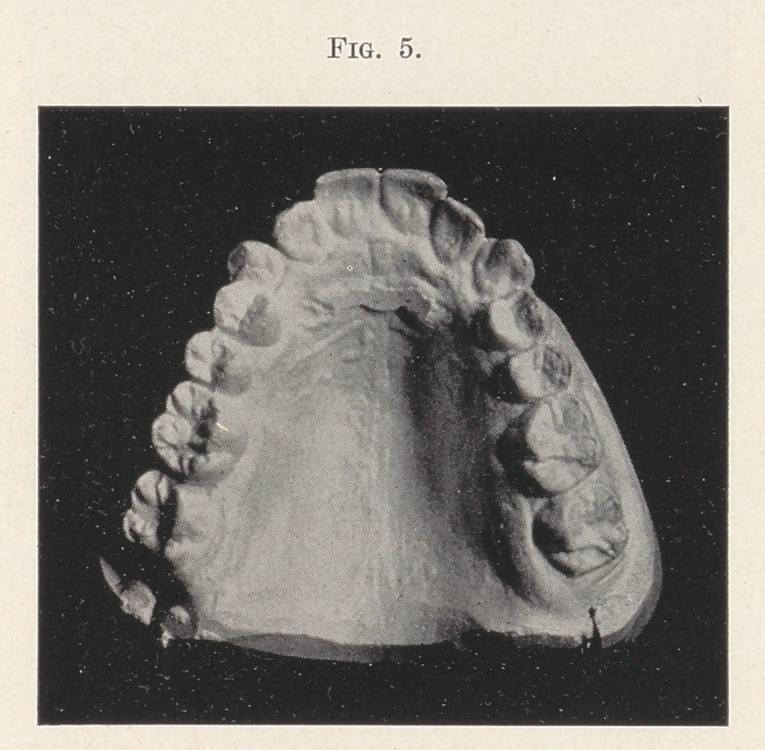


**Fig. 6. f6:**
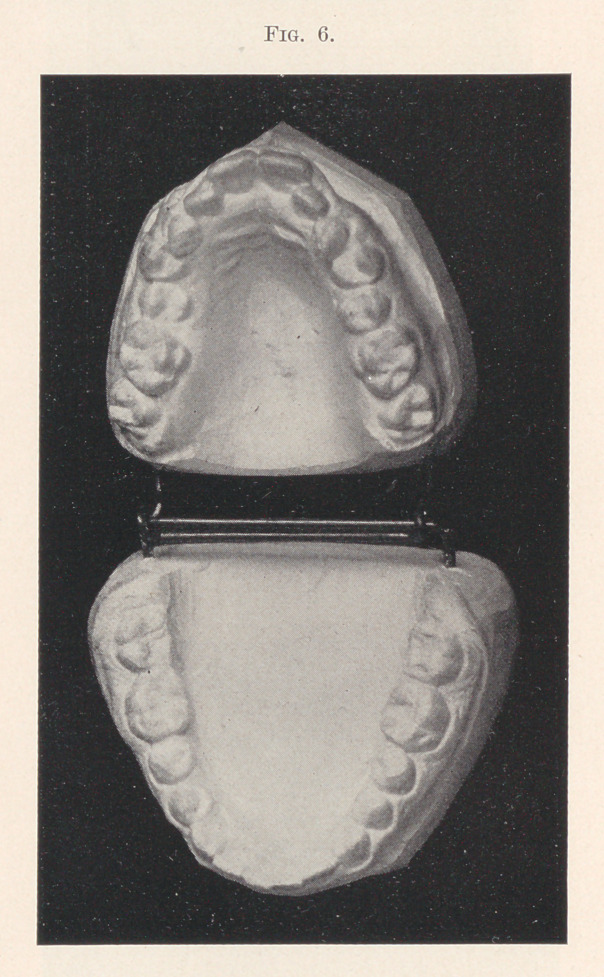


**Fig. 7. f7:**
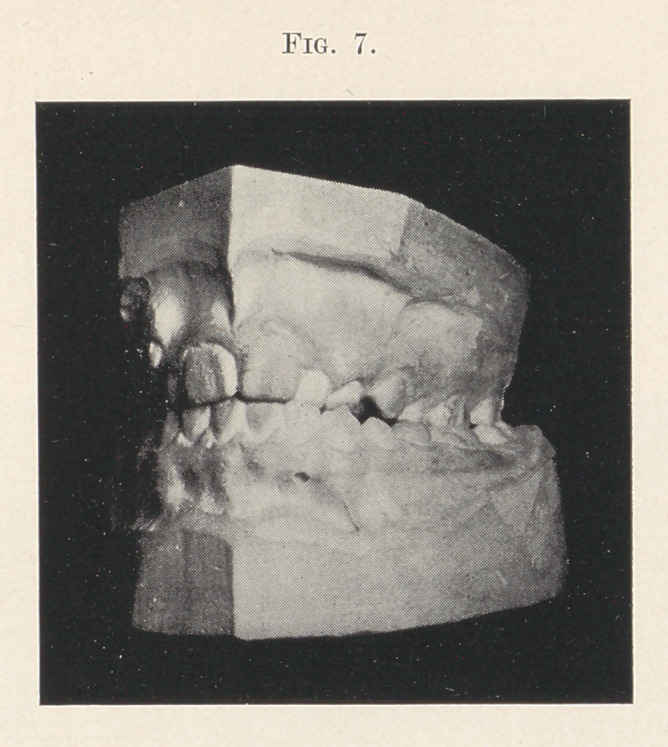


**Fig. 8. f8:**
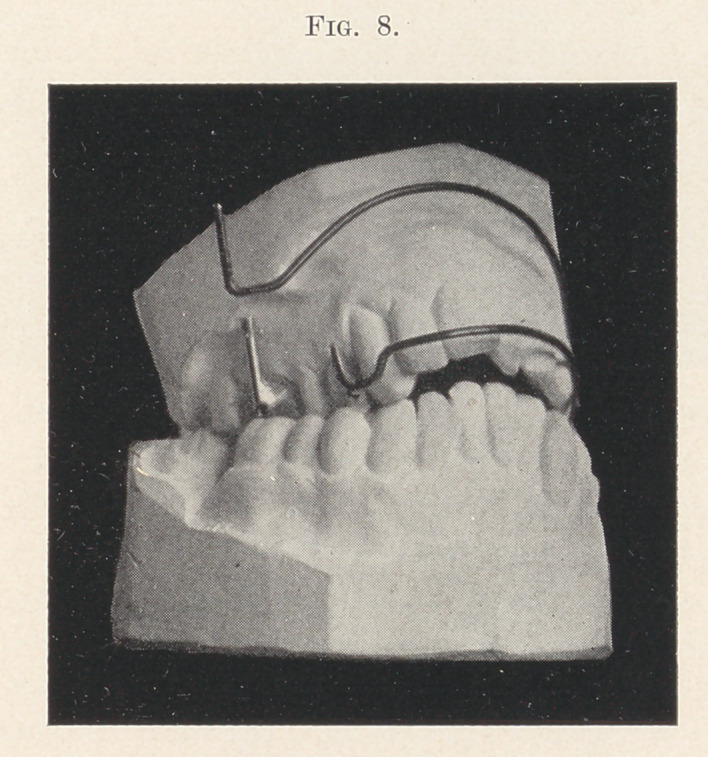


**Fig. 9. f9:**
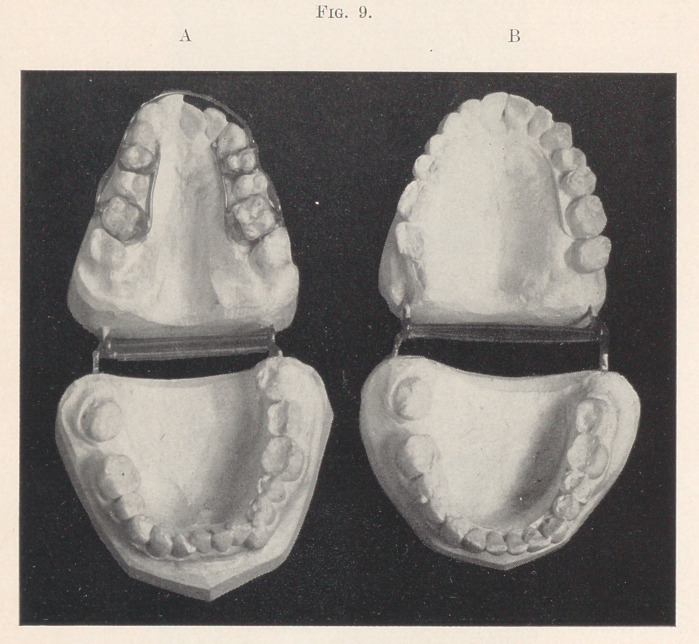


**Fig. 10. f10:**
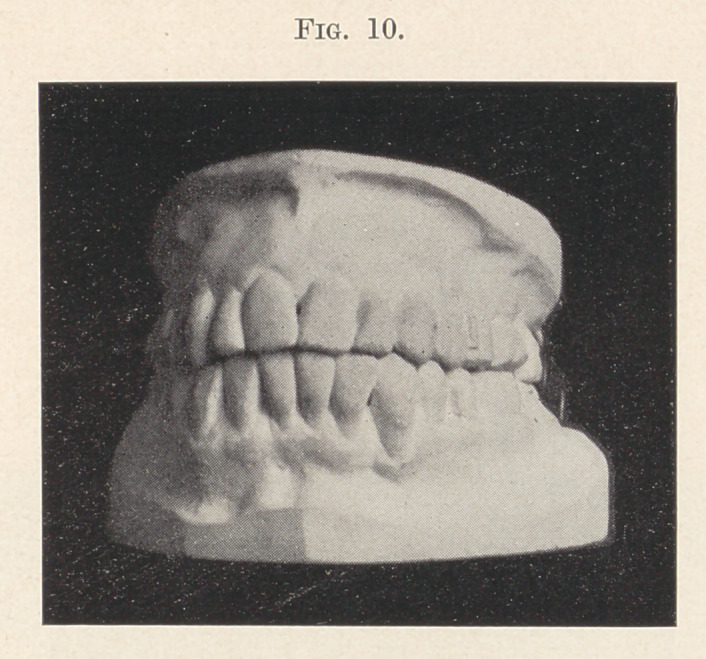


**Fig. 11. f11:**
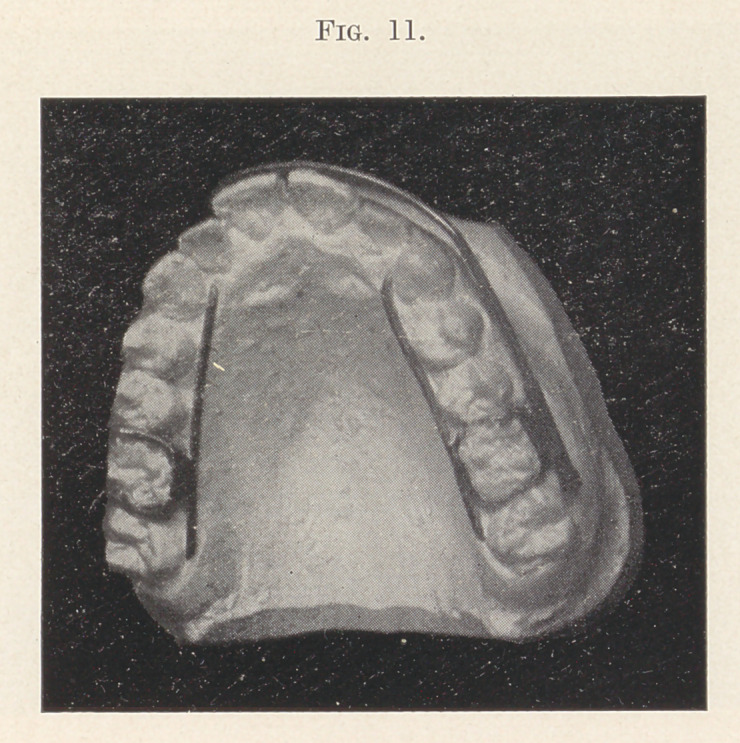


**Fig. 12. f12:**
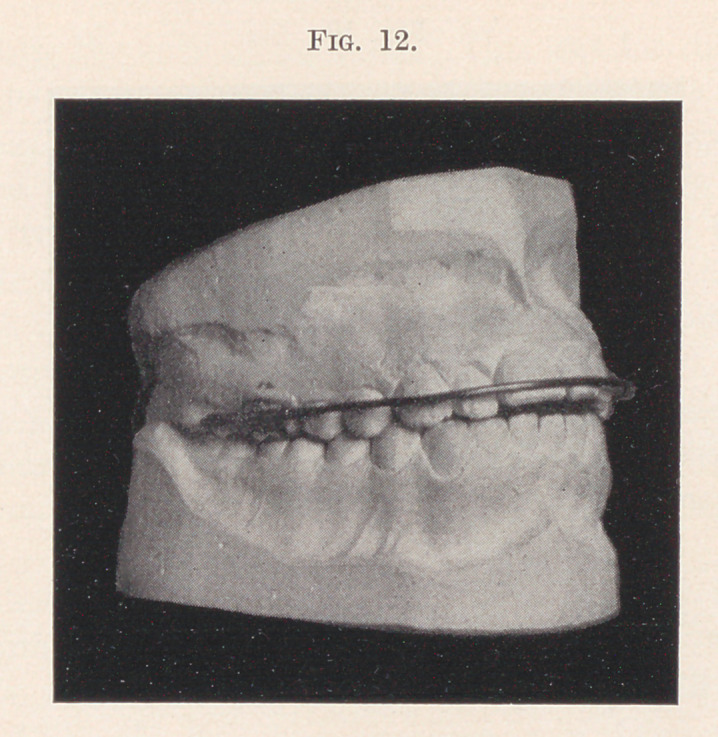


**Fig. 13. f13:**
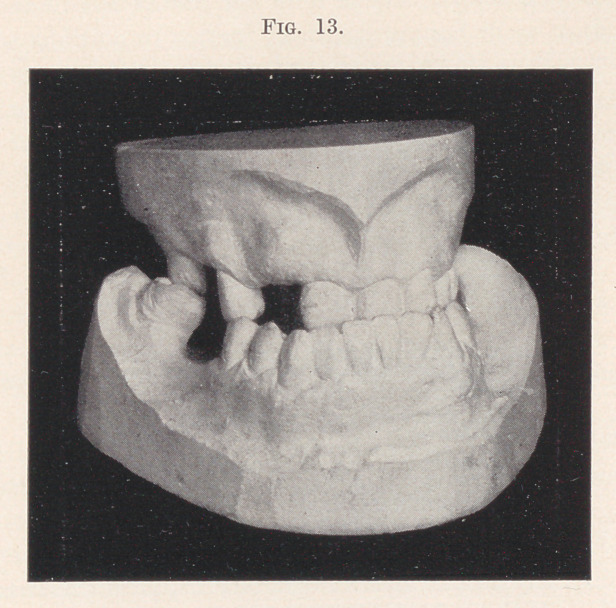


**Fig. 14. f14:**
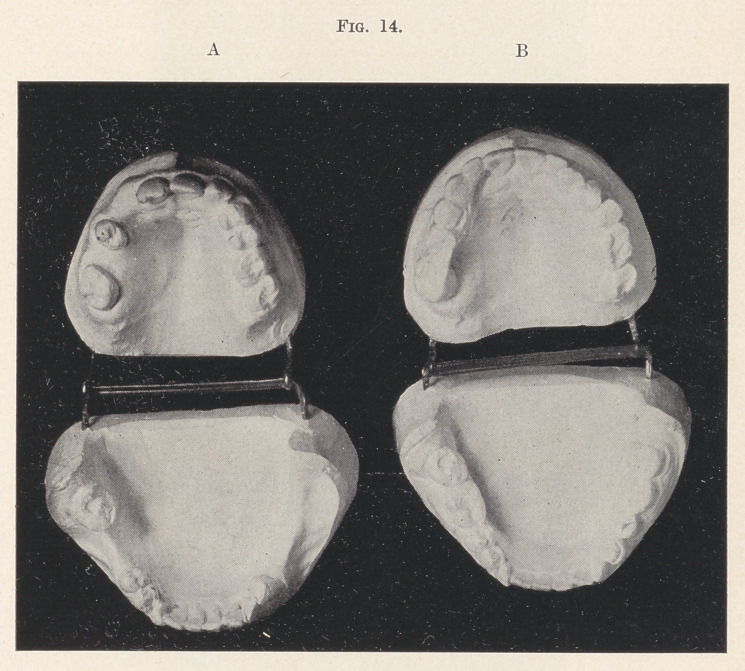


**Fig. 15. f15:**
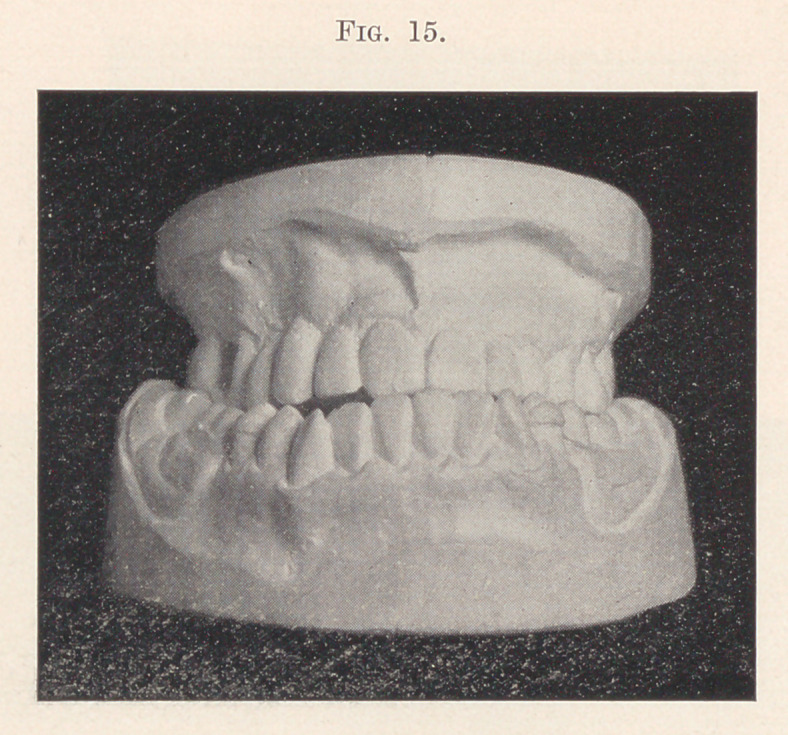


**Fig. 16. f16:**
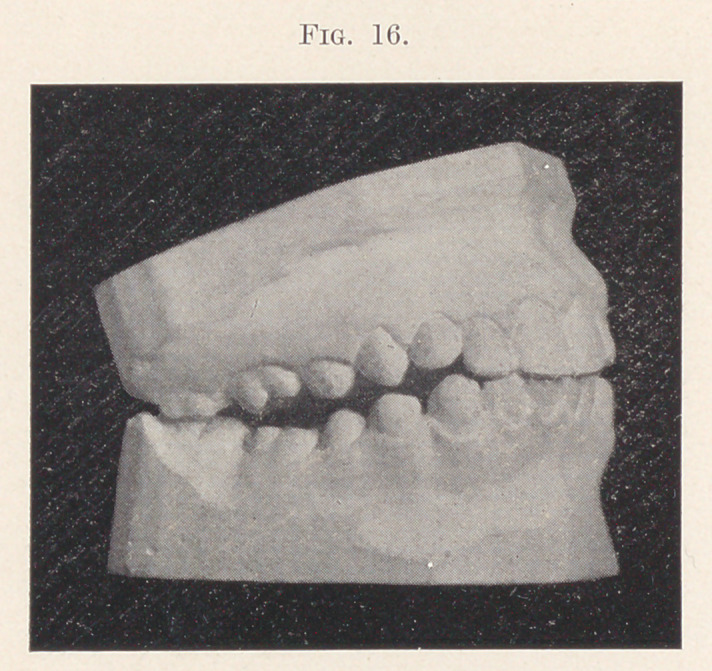


**Fig. 17. f17:**
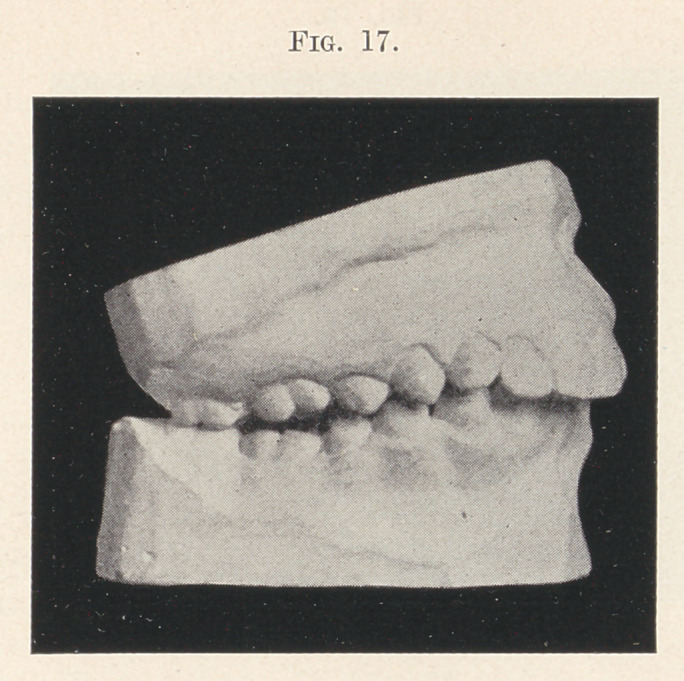


**Fig. 18. f18:**
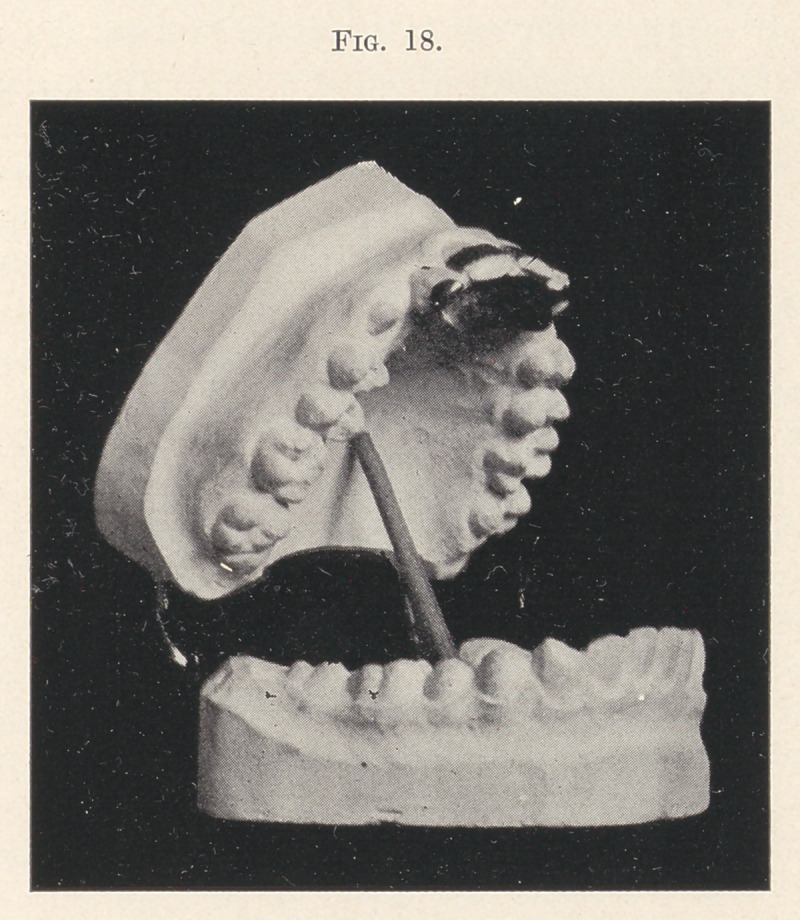


**Fig. 19. f19:**
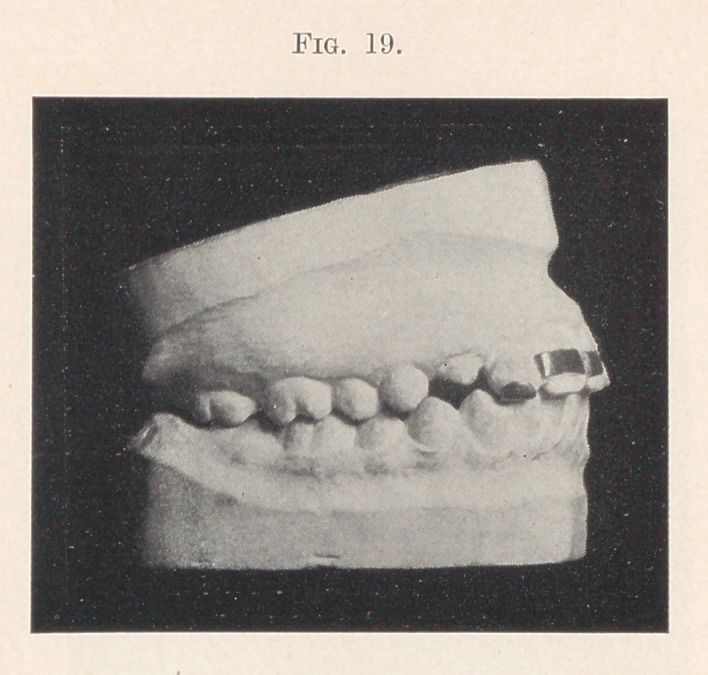


**Fig. 20. f20:**
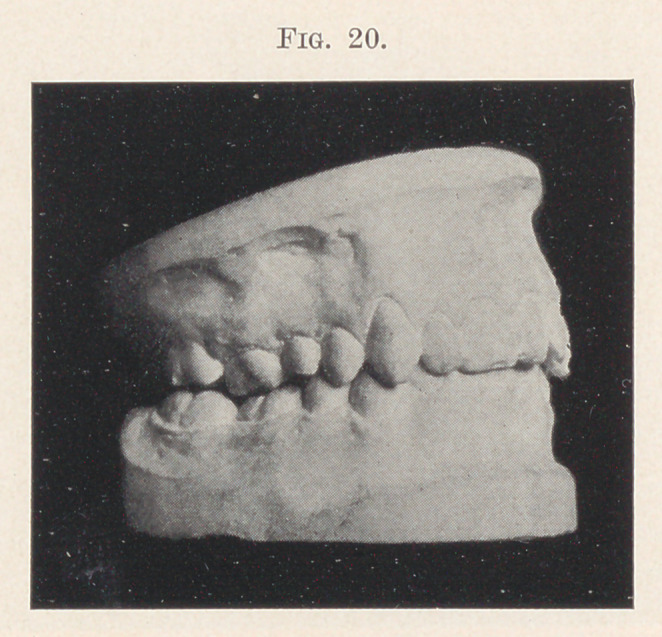


**Fig. 21. f21:**
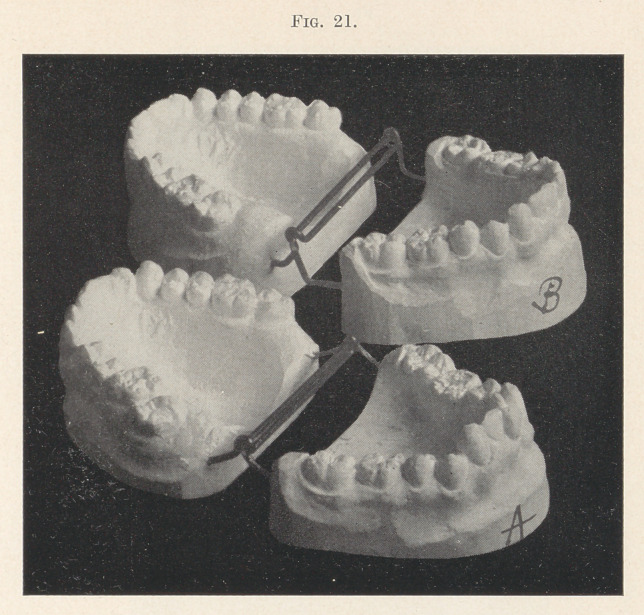


**Fig. 22. f22:**
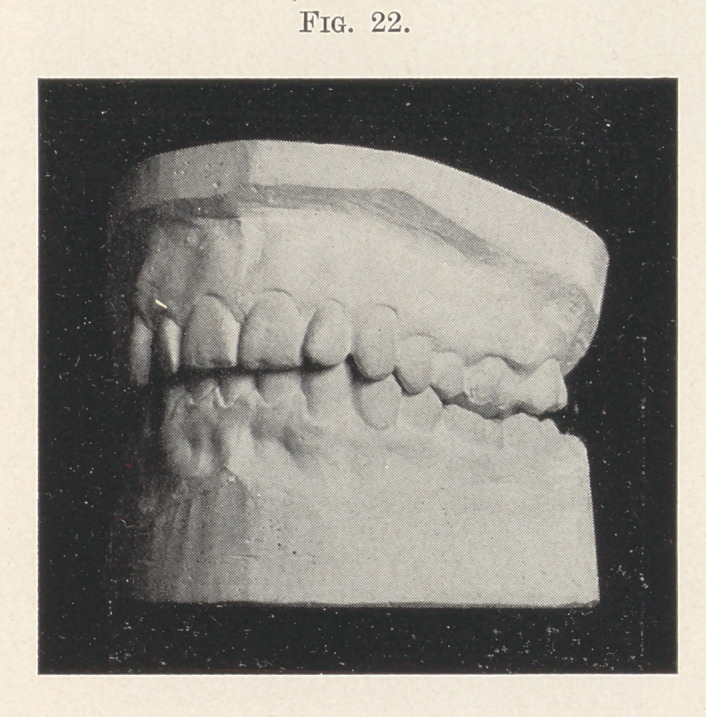


**Fig. 23. f23:**
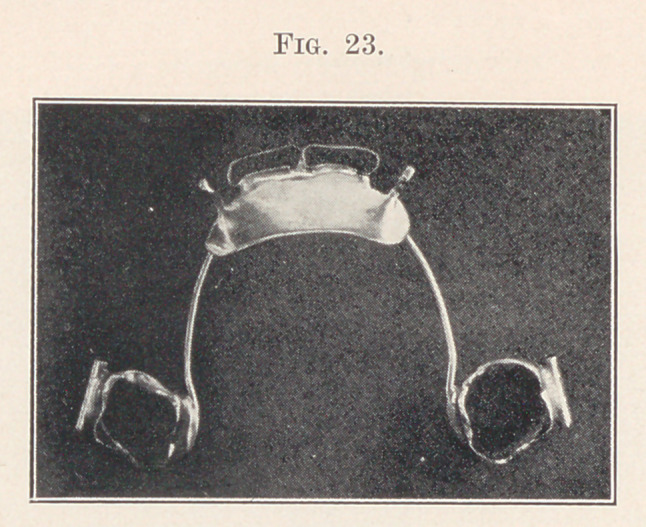


**Fig. 24. f24:**
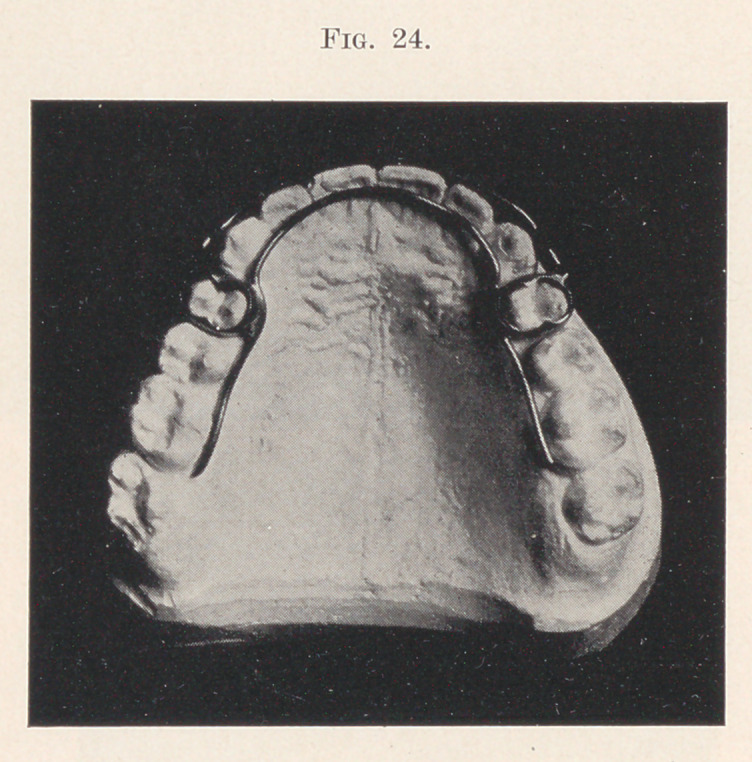


**Fig. 25. f25:**